# Lacrimal Proline Rich 4 (LPRR4) Protein in the Tear Fluid Is a Potential Biomarker of Dry Eye Syndrome

**DOI:** 10.1371/journal.pone.0051979

**Published:** 2012-12-18

**Authors:** Saijyothi Venkata Aluru, Shweta Agarwal, Bhaskar Srinivasan, Geetha Krishnan Iyer, Sivakumar M. Rajappa, Utpal Tatu, Prema Padmanabhan, Nirmala Subramanian, Angayarkanni Narayanasamy

**Affiliations:** 1 Biochemistry and Cell Biology Department, Vision Research Foundation, Sankara Nethralaya, Chennai, Tamilnadu, India; 2 Ocular Surface Clinic, Medical Research Foundation, Sankara Nethralaya, Chennai, Tamilnadu, India; 3 Cerebrovascular and Vasculitis Research Foundation, Chennai, Tamilnadu, India; 4 Biochemistry Department, Indian Institute of Science (IISc.), Bangalore, Karnataka, India; 5 Oculoplasty Department, Medical Research Foundation, Sankara Nethralaya, Chennai, Tamilnadu, India; Wayne State University School of Medicine, United States of America

## Abstract

Dry eye syndrome (DES) is a complex, multifactorial, immune-associated disorder of the tear and ocular surface. DES with a high prevalence world over needs identification of potential biomarkers so as to understand not only the disease mechanism but also to identify drug targets. In this study we looked for differentially expressed proteins in tear samples of DES to arrive at characteristic biomarkers. As part of a prospective case-control study, tear specimen were collected using Schirmer strips from 129 dry eye cases and 73 age matched controls. 2D electrophoresis (2DE) and Differential gel electrophoresis (DIGE) was done to identify differentially expressed proteins. One of the differentially expressed protein in DES is lacrimal proline rich 4 protein (LPRR4). LPRR4 protein expression was quantified by enzyme immune sorbent assay (ELISA). LPRR4 was down regulated significantly in all types of dry eye cases, correlating with the disease severity as measured by clinical investigations. Further characterization of the protein is required to assess its therapeutic potential in DES.

## Introduction

Dry eye syndrome (DES), an ocular sicca syndrome is a disorder of the tear film that results in epithelial cell damage and disruption of the normal homeostasis at the ocular surface [1]. The prevalence as per the recent study in US is reportedly 12% in men and 22% in female above 50 years of age. DES is found to be associated with systemic diseases especially diabetes mellitus and cardiovascular disease [2]. The prevalence in India is based on a report from a tertiary based hospital study, which showed overall prevalence of 29% with preponderance in women (27%) as against men (12%) [3]. Thus, there seems to be a high prevalence of this disease worldwide.

Tear film plays crucial role as a protective barrier of the eye and has other key functions such as nutrition, lubrication and optical refraction [4]. Tears are composed of mucins, lipids, proteins, electrolytes and various other metabolites which are involved in various functions like ocular surface wound healing, antimicrobial and anti-inflammatory activities, apart from ensuring the surface integrity of the cornea [5], [6], [7], [8], [9], [10], [11], [12]. The major tear proteins include lysozyme, lactoferrin, secretory immunoglobulinA (sIgA), lipocalin, albumin and lipophilin and the tear protein content varies from 6 to 10 mg/ml [13], [14]. Changes in tear protein profile have been shown to be associated with various systemic and pathological conditions such as in diabetes, fungal keratitis and blepharitis [10], [15], [16]. Since pathological processes can be described as aberrations in the homeostasis of protein function, protein profiling using proteomic approaches will aid in detecting the differentially expressed disease specific biomarkers. Tears are being recently considered as a valuable specimen for analysis, as it is available by non-invasive procedures.

In this study we looked for the differentially expressed proteins in tear samples of DES using a 2D electrophoresis based proteomic approach, with peptide identification by mass spectrometry. One of the differentially expressed protein namely lacrimal proline rich 4 protein (LPRR4) characteristic of tear was evaluated as a potential biomarker. Proline-rich proteins (PRPs) are highly polymorphic and belong to a class of intrinsically unstructured proteins. Proline-rich domains in protein are known to act as flexible regions that binds rapidly and reversibly as they provide the binding sites for the specific interacting partners [17]. The tissue-specific synthesis such as the salivary PRP is constitutively expressed in humans [18], [19]. The three major functions of salivary PRPs are to act as inhibitors of calcium phosphate precipitation, bind and clear potential bacterial pathogens as well as binding to minerals or tannins [20]. A truncated form of lacrimal proline-rich protein in the tear was reported by Fung KY et al [21]. A quantitative measure of the tear levels of the protein LPRR4 is reported in this study.

## Materials and Methods

### Materials

DIGE minimal Cydye labeling kit (GE healthcare,UK), Tris, Urea, CHAPS, DTT, Iodoacetamide, Acrylamide, Bisacryamide, pH 3–10, 17 cm IPG strips (Bio-Rad Laboratories, USA), 3 kDa cutoff filters (Amicon – Millipore, USA), chemicals for Phosphate buffered saline (pH:7.4) (Merck, India), Protease inhibitor cocktail (Sigma USA), Schirmer strips, (Conta care, Baroda, India), and Bradford kit for protein estimation (Pierce, USA), Ammonium bicarbonate (Merck, India), Acetonitrile (Merck HPLC grade), Formic acid (Fluka, USA), sequencing grade trypsin (promega, USA) ELISA kit for LPRR4 (USCN, China) were used in the study.

### Ethics

The study was approved by institutional ethical committee and also adheres to the guidelines of Helsinki declaration. The tear samples were collected after written informed consent using sterile Schirmer strips.

### Exclusion Criteria

The exclusion criteria included, as those who were less than 18 years, cases with history of surgical intervention, chemical injury, complaints of ocular pain or discomfort and any recent history of ocular diseases, those on contact lens wear, connective tissue diseases (other than Rheumatoid arthritis ), diabetes mellitus and Parkinson’s disease.

### Dry Eye Diagnostic Criteria

Diagnosis of DES and the grading of the severity is based on various clinical parameters such as Schirmer’s test (< than 10 mm for 5 minutes, without anesthesia), tear breakup time (TBUT) (<10 sec), corneal and conjunctival staining score based on Dry Eye Work Shop study (DEWS) [22] as well as using MacMonnies questionnaire [23]. A comprehensive clinical proforma was used to document the clinical details given in the Table S1. The severity of DES was based on the grading done using the clinical parameters.

### Tear Sample Details

As a prospective age and sex matched case- control study, 73 controls (mean age: 43±12 y, 30 M, 43 F) and 129 DES (mean age: 45±3 y, 51 M, 78 F) were recruited to look for the differentially expressed proteins. Of these, 2D gel electrophoresis was done in 39 healthy controls (mean age: 43±12, 12 M, 27 F), 26 cases of Non Sjogren’s (NS) (mean age: 40±17 y, 10 M,16 F ), 15 cases of primary Sjogrens (PSS) (mean age: 48±11 y, 5 M, 10 F), 26 cases of dry eye secondary to Rheumatoid arthritis (RA), (mean age: 48±10 y, 6 M, 20 F). DIGE was done in 18 controls (mean age: 43±12 y, 8 M, 10 F), 11 cases of NS (mean age: 42±16 y, 7 M, 4 F); 8 cases of PSS, (mean age: 46±11 y, 4 M, 4 F), 16 cases of dry eye secondary to RA, (mean age: 49±8 y, 2 M, 14 F). Further, for the quantification of LPRR4 by ELISA, tear samples were prospectively collected from dry eye cases (mean age: 49±16 y, n = 27 ) associated with NS (mean age: 45±20 y, n = 9), PSS (mean age: 49±20 y, n = 7) and RA (mean age: 52±9 y, n = 11) with age matched controls (mean age: 43±10 y, n = 16, 10 M, 6 F).

### Collection of Tear Specimen

Tears were collected using sterile Schirmer strips by making the person seated in a comfortable posture with raised head, against any direct source of light or flow of air. The Schirmer strip was then placed in the lower cul-de-sac region and was allowed to absorb the tear for 5 min in open eye condition. The strip was then placed in sterile vial at −70°C until processing. While using Schirmer’s the tear collected is considered as reflex tear as it was collected with no local anesthesia [24].

### Tear Protein Extraction for 2D Electrophoresis (2DE)

The tear absorbed on to the strip was then placed in sterile vial, immediately stored at −80°C until processing. For 2DE, the tear protein was extracted using 8 M Urea Buffer containing 3% CHAPS and 25 mM DTT (pH: 7.4) [25] and for DIGE the tear protein was extracted using 30 mM Tris–HCl buffer containing with 8 M urea, 3% CHAPS and 0.5 mM TCEP (pH: 8.5). During extraction 300 µl of buffer was added to the strip, with 30 µg of protease inhibitor cocktail and after vortexing briefly, was left at 4°C for 3 hours. At the end of 3 hours time, the vial was centrifuged at 5000 rpm for 10 min at 4°C and the supernatant was subjected to desalting using 3 kDa cutoff filters. Protein estimation was done using Bradford assay. 30 µg protein was used for both 2DE and DIGE. The proteins that were either down regulated or up regulated were considered significant if the density variation was more than 2 fold and was observed in more than 50% of the cases or controls.

### 2D Differential Gel Electrophoresis (2D-DIGE)

For 2D-DIGE, tear proteins from controls and Dry eye subjects were pooled. Each of the control and dry eye used was a pool of 3 specimens in each group. 14 such sets were subjected to DIGE analysis, in which the DES were from primary Sjogren (2 sets), DES secondary to RA (5sets) and Non Sjogren (7sets). 30 µg protein from each control and DES was used for Cy 3 and Cy 5 labeling. 15 µg protein from each group was used for Cy 2 labeling as internal standard. Thus, samples from either dry eye or healthy control were labeled with Cy3 or Cy5 cyanine dyes using 30 µg protein, while 15 µg protein was used for labeling with internal standard samples with Cy2 dye. 240 pmol of Cy dye in 1 µL of anhydrous N, N dimethylformamide (DMF) per 30 µg of protein was used. Labeling of protein with Cy dye was done according to the manufactures instructions (GE Health care, UK).

Scanning of the gels for 2DE was done using GS 800 densitometry and the quantitative analysis done using PD Quest software. For DIGE scanning was done using typhoon scanner (GE Health care, UK) with 500 V PMT, 100 microns pixel. The laser wavelengths for each Cy dyes are Cy3 Ex 532 Em 580 nm, Cy 5 Ex 633 Em 670 nm and for Cy 2 Ex 488 Em 520 nm. Gels were analyzed using Decyder 2D version 7.0 software (GE Health care, UK).

### Mass Spectrometric Analysis of Spots

Protein spots were excised from the gel, subjected to in gel tryptic digestion, analyzed by mass spectrometry using nano LC-MS/MS as detailed earlier [25]. Briefly the silver stained spots was excised from the gel, destained and reduced using 100 mM DTT at 56°C for one hour followed by alkylation with 55 mM iodoacetamide for 45 min at RT. Digested the proteins with trypsin (12.5 ng/µl in NH_4_HCO_3_) for 12–14 h, centrifuged the gel pieces, stored the supernatant. Extraction of the peptides was done using 50% acetonitrile +5% formic acid mixture and dried by speed vac. For MS analysis, 2% acetonitrile and 0.2% formic acid was used to reconstitute the peptides.

### Nano LC – MS/MS Analysis of Tear Proteins

Peptide mixtures were loaded on to a nano LC reverse phase column of internal diameter 75 µm, packed with C18 particles of size 5 µm (Michrom) and eluted into a ESI – Quadra pole Time of Flight Mass Spectrometer (Q STAR Elite, MOS, Geiex – Applied Biosystems) with a 60 min gradient. Fragments ion spectra were recorded using information dependent acquisition (IDA). Data was analyzed using Protein pilot 2.0 Software with All Entries Database.

### ELISA for Lacrimal Proline Rich 4 Protein

To quantitate the identified protein, the protein extraction from Schirmer’s strip was done using 300 µl PBS with protease inhibitors, incubated at 4°C for 3 h with intermittent mixing. Further, the solution was centrifuged at 5000 rpm for 10 min at 4°C, supernatant was stored at –80°C until processing. The levels of LPRR4 were estimated using ELISA kit from USCN life science Inc., as per the manufacturer’s instructions. The microtiter plate provided in the kit has been pre-coated with an antibody specific to LPRR4. Standards or samples are then added to the appropriate microtiter plate wells with a biotin-conjugated antibody preparation specific for LPRR4. Avidin conjugated to Horseradish Peroxidase (HRP) is the enzyme substrate based detection used and the color change is measured spectrophotometrically at a wavelength of 450 nm in the tear samples to using the LPRR4 standard graph and calculated in ng/ml and expressed as µg/ml of tear volume. To arrive at the tear volume, a known volume of tear collected using capillary from control was calibrated using Schirmer’s strip for the wetness in mm. Accordingly 1 µL of capillary tear is ≡ 1.5 mm in the Schirmer’s strip.

### mRNA Expression of LPRR4 Using Reverse Transcriptase Polymerase Chain Reaction (RT-PCR)

LPRR4 mRNA expression in lacrimal gland tissue of human was studied. The expression of LPRR4 was also evaluated in other ocular tissue namely human corneal epithelial tissue for comparison and to evaluate the tissue specificity. The lacrimal gland tissue was obtained during surgical procedure from the patients who underwent ptosis correction and the corneal epithelium was obtained from the myopic patients who underwent epilasik procedure for refraction correction after an informed consent of the patient which was approved by the institutional research board. RNA was extracted from the tissues using TRI reagent method, cDNA conversion was done from RNA using iScript™ cDNA synthesis kit (Bio-Rad, Herclus,CA) and Reverse transcriptase PCR (GeneAmp PCR system 9700 from Applied Biosystems) was done for LPRR4. 2 µg of RNA was used for the cDNA conversion. 200 ng of cDNA was used for PCR for all the samples. The Primers used for LPRR4 were designed using genscript website. Forward primer sequence 5′TGCTCTCAGTGGTCCTTCTG3′ and Reverse primer sequence 5′CTTCAGGAGGAGGTCTCTGG 3′, the product base pair size was 144 bp. The negative control had all reagents except the cDNA. The PCR conditions used were : Initial denaturation temperature of 94°C/5 min, 94°C –1 min, annealing temperature of 57°C/1 min and extension temperature of 72°C/1 min for 30 cycles with final extension at 72°C/7 min and then at 4°C.

### Statistical Analysis

Students‘t’ test was used to assess the statistical significance of the data obtained. *P* value <0.05 was considered significant. Pearson’s correlation coefficient was calculated using SPSS version 14.1(Ilinois, USA). For DIGE statistical analysis, the Biological Variance Analysis (BVA) of the peptide spots in the DIGE gels was done by one way ANOVA using Decyder software version 7. *p value* <0.05 was considered significant.

## Results

**Figure 1:**
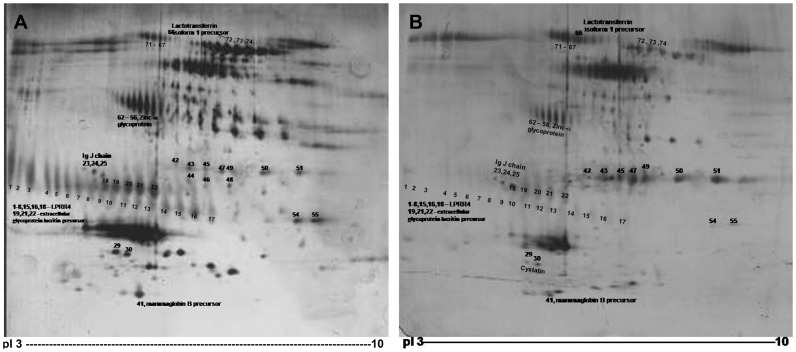
2D gel map of tear protein A: Control. B: Dry eye syndrome. Tear protein (30 µg) separated on 17 cm, pH 3–10 IPG strip in the first dimension and 13% SDS-PAGE in the second dimension. The differentially expressed proteins in DES compared to control are numbered.

The tear samples in control and DES were subjected to 2D electrophoresis, and the differentially expressed peptide spots were analyzed by densitometry analysis using PD Quest software (Figure 1). 56 peptide spots were found to be differential in DES compared to the control. Amongst these, the 30 peptide spots corresponding to 6 proteins were identified by Mass spectrometry (Table 1). LPRR4, a lacrimal gland specific protein that was down regulated in >95% cases of DES by more than 2 fold, was chosen for further validation as not much is known on this protein. Table 2 shows the down regulation of LPRR4 in all types of DES, namely primary Sjogren’s, non Sjogren’s including Steven Johnson’s syndrome and secondary to rheumatoid arthritis based on the PD Quest analysis of the detected peptide spots in the 2D gels. The extent of decrease in the intensity of the spot is found to be >75% in all the types of DES (Table 2).

**Table 1:** Differentially expressed tear proteins identified by LC-MS/MS.

Spot No.	Mean spot volume intensity	% cases showing differential expression	Name of theidentifiedprotein	Accession No.	Peptide sequence identified	% coverage of peptide
1–8, 15, 16	2.6 ↓	99%	LPRR4	gi|15444886	FPSVSLQEASSFFQR(1 Peptide)	37%
9–14	2.5 ↓	90%	Not identified	–	–	–
21, 22	2.4 ↓	95%	Lacritin precursor	gi|54607120	DGAGDVAFIRLADFALLCLKLRPVAAEVYGTER(3 peptides)	25%
18, 19	2.1 ↓	95%	Extracellular glycoprotein lacritin precursor	gi|15187164	SILLTEQALAK(1 Peptide)	26%
23–25	1.8 ↓	100%	Immunoglobulin J	gi|21489959	SSEDPNEDIVERCYTAVVPLVYGGEY(2 peptides)	31%
42–51	2.0 ↑	60%	Not identified	–	–	–
54,55	1.9 ↓	70%	Not identified	–	–	–
29,30	2.0 ↓	85%	Cystatin*	–	–	–
40,41	2.3 ↑	60%	Mammagobulin Bprecursor	gi|4505171	ELLQEFIDSDAAAEAMGTINSDISIPEYKQCFLNQSHR(3 peptides)	43%
56–62	2.5 ↓	100%	Zn-alpha-glycoprotein	–	–	–
67–71	1.9 ↓	90%	Not identified	–	–	–
72–74	2.1 ↓	90%	Lactotransferrin isoform 1 precursor Isoform 2	gi|54607120gi|312433998	DGAGDVAFIRDGAGDVAFIR(1 peptide)	17%
75, 76	2.5 ↓	80%	Not identified	–	–	–

* From literature.

Tear proteins were profiled by 2D electrophoresis. A total of 56 peptides showed differential expression. 30 peptide spots corresponding to 6 proteins namely, lacrimal proline rich 4 protein (LPRR4), immunoglobulin J, cystatin, Zinc alpha glycoprotein, lacritin precursor, extracellular glycoprotein lacritin precursor, lactotransferrin isoform 1 and 2, mammaglobulin B precursor. The rest of the spots are not yet identified.

**Table 2:** Down regulation of LPRR 4 protein in various types of DES cases as determined by 2D electrophoresis and PDQuest analysis.

Name	DES with Primary Sjogren’s(n = 15)	DES with Non Sjogren’s(n = 26)	DES secondary to RA(n = 26)
**% cases** **down regulated**	**100**	**100**	**96**
**% ↓ spot intensity**	**75**	**80**	**75**

**Figure 2:**
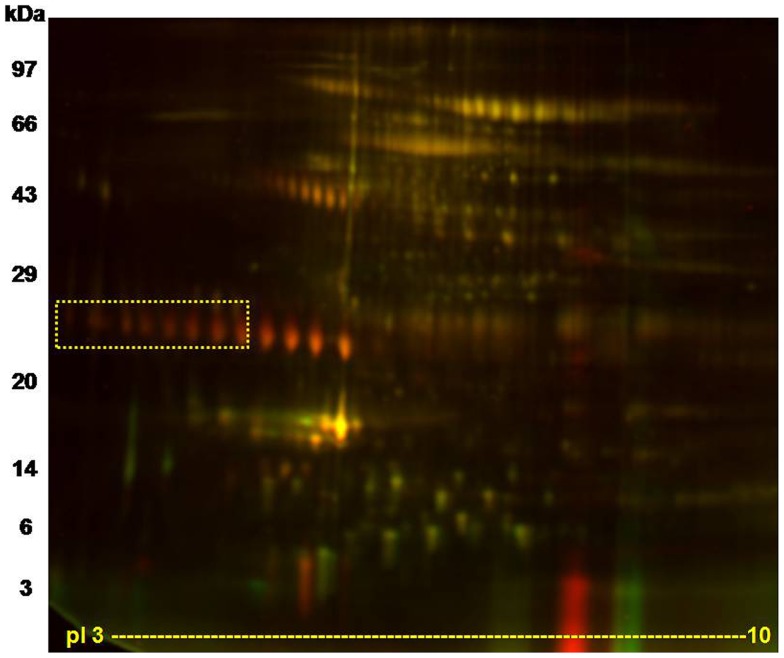
A representative DIGE image showing the tear protein profile. 8 peptide spots identified as LPRR4 that was down regulated in Dry eye condition are shown within the square box. DES case and control tear protein (30 µg) were labeled with Cy5 and Cy3 respectively as described in methods section. The range of the horizontal dimension is isoelectric point (from pI 3 to 10) using 17 cm IPG strips; the range of the vertical dimension is molecular weight (from approx. 97 to 3 kDa) on a 13% SDS-PAGE.

**Figure 3:**
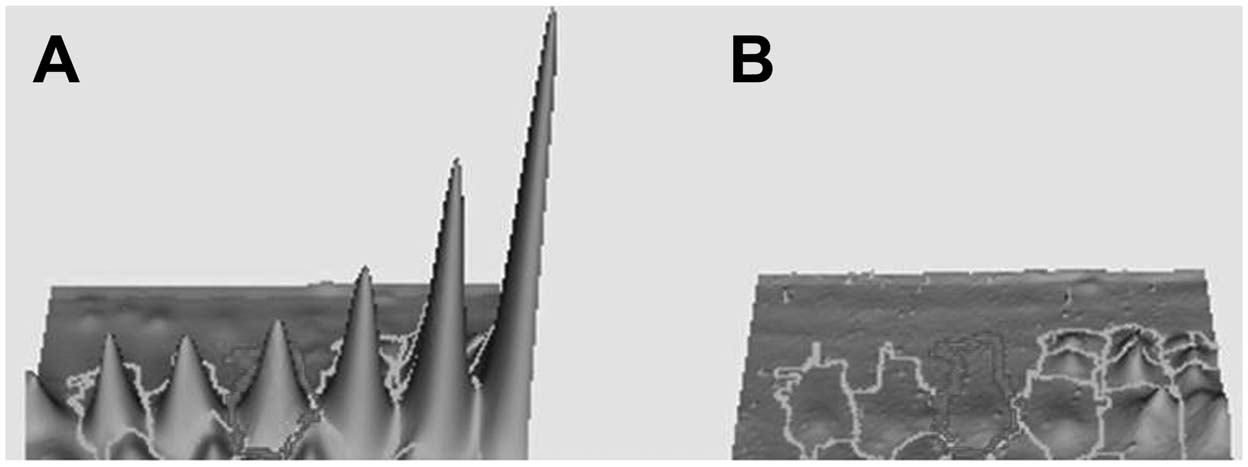
3D DeCyder image of LPRR4 after DIGE. Each protein in 3D view is shown. The 3D peak of each protein was generated based on the pixel intensity versus pixel area, normalized by the peak area of standard (Cy-2-labeled). A: control B: DES. DES showed a significant reduction of these five peptides identified as LPRR4 *(p = 0.009)* as observed by BVA analysis using Decyder software.

DIGE profile showed a 4 fold decrease of LPRR4 protein in dry eye as per the spot volume ratio calculated using Decyder 2D 7 software (Figure 2). Figure 3 shows the representative 3D view of LPRR4 peptide revealing the down regulation of LPRR4 protein based on the peak area. DES showed a significant reduction of these five peptide spots identified as LPRR4 *(p = 0.009)* as observed by BVA analysis using Decyder software.

**Figure 4:**
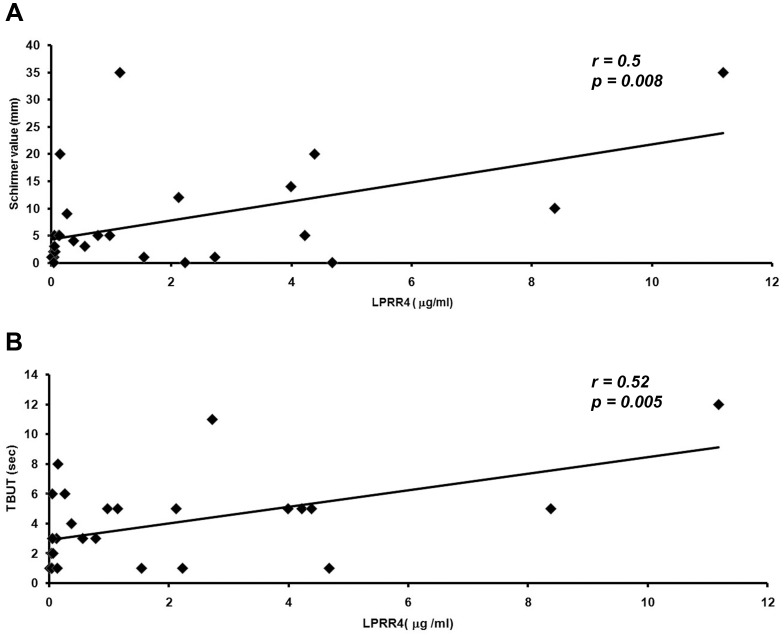
Pearson’s Correlation graph of LPRR4 levels in Dry eye syndrome with clinical parameters namely Schirmer’s value and Tear Breakup Time (TBUT). A. Tear LPRR4 levels vs Schirmer value (*p = 0.008),* B. Tear LPRR4 levels vs TBUT. (*p = 0.005*).

**Figure 5:**
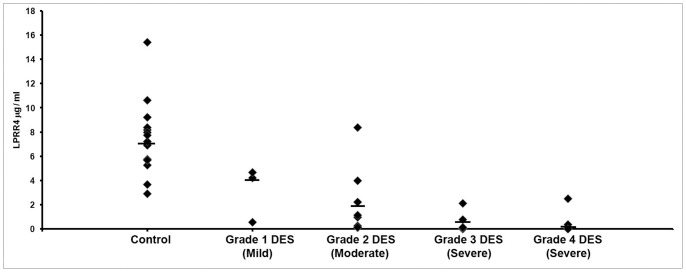
Distribution graph of LPRR4 levels based on Dry eye syndrome (DES) grade, grade 1(mild DES) to grade 4 (severe DES). A shift in the median was observed compared to the control.

Table 3 shows the levels of LPRR4 protein in the tear as detected by ELISA with a significant decrease in the levels of the protein in all types of DES. A mean LPRR4 level of 6.9±0.78 µg/ml with a range of 2.9 to 15.4 µg/ml range was observed in the normal tear and it was found to be decreased by 4.6 fold to 1.5±0.52 µg/ml in the DES cases with a range of 0.032 to 11.2 µg/ml in DES cases (Table 3). A significant positive correlation between the levels of the LPRR4 protein and the Schirmer’s value (r = 0.55 & *p = *0.008) as well as with that of the TBUT values (*r* = 0.52 & *p* = 0.005) were observed, indicating the correlation of the protein levels with the severity of dry eye (Figure 4). Figure 5 shows the distribution graph of the LPRR4 in control and different grades of DES wherein there is clear shift of the median with disease progression as measured in DES grade.

**Table 3:** LPRR4 levels in tear from various types of DES by ELISA.

Parameter	Control(n = 19)	Total DES cases(n = 27)	DES with Non Sjogren’s(n = 10)	DES with primary Sjogren’s(n = 7)	DES Secondary to RA(n = 10)
**LPRR4** **µg/ml**					
**Mean**	6.95	1.5	1.99	2.45	0.44
**SEM**	0.78	0.52	0.64	0.94	0.14
***P value***		<0.001	0.001	0.007	<0.001
**% severe cases**		44%	20%	43%	70%

**Figure 6:**
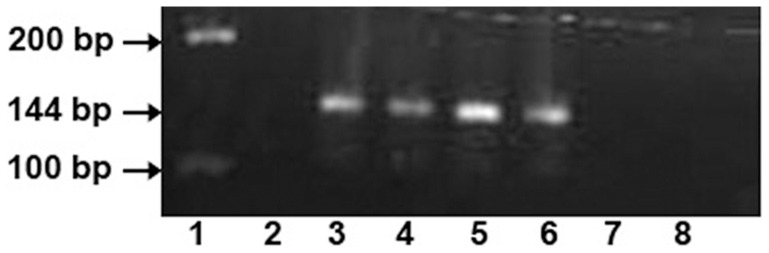
mRNA expression of LPRR4 in human lacrimal gland tissue as 144 bp product using RT-PCR showing tissue specificity for LPRR4. Lane 1∶100 and 200 bp ladder, Lane 2: Negative control (except cDNA), Lane 3–6: human lacrimal gland tissue (2 µg), Lane 7–8: human corneal epithelial tissue ((2 µg).

mRNA expression of LPRR4 was observed in human lacrimal gland specifically, while the human corneal epithelial tissue did not show the expression which indicates the tissue specificity of this protein (Figure 6).

## Discussion

Human tears contain large number of proteins exerting significant influence on tear film stability, ocular surface integrity, and visual function. Proteins secreted by the lacrimal glands has been shown to contribute to the dynamics of the tear film in both health and disease [26]. The possible mediators of lacrimal gland insufficiency in DES includes increased levels of pro inflammatory cytokines, production of auto antibodies, apoptosis, alterations in signaling molecules, hormonal imbalance and many others [27], [28], [29]. Therefore alterations in the proteins profile are indicative of the disease mechanism and identification of marker protein can give clues on the disease severity as well as on the underlying pathology.

Proteomic study using mass spectrometric analysis to identify protein biomarkers, further linking it to the disease activity as well as the treatment responses are plenty in number. However there are limited studies using tear as a specimen to identify such biomarkers. Normal production of tear proteins, such as lysozyme, lactoferrin, lipocalin, and phospholipase A2 beta-2 microglobulin, is reportedly decreased in keratoconjunctivitis sicca [12]. Selective defect in aquaporin 5 (AQP5) trafficking is seen in patients with sjogren’s syndrome [30]. Lipophilin was shown to be significantly increased in the dry eye relative to the normal as studied in rabbit model [31]. Most of these proteins are high abundant proteins of the tear. The subtle changes in the low abundant and low molecular weight proteins need attention and needs robust protocols to address the same. In this study, no pooling of samples has been done as in most of other studies. Except for the 3 kDa cutoff filtration, no other sample enrichment protocols that can result in loss of proteins was used. This study has shown that tear can be a valuable specimen to pick up biomarkers of dry eye syndrome using a proteomic approach in a non invasive manner. Addition of tear specific protein biomarkers can be valuable in the treatment of dry eye syndrome.

Among the differentially expressed proteins identified by mass spectrometry, namely, LPRR4, Lacritin precursor, extracellular glycoprotein lacritin precursor, Immunoglobulin J, mammaglobulin B precursor, lactotransferrin isoform 1 precursor and isoform 2, 4 of them namely LPRR4, lacritin precursor, extracellular glycoprotein lacritin precursor, Ig J showed down regulation in more than 95% of the cases. Of these, the proteins LPRR4, Lacritin precursor, extracellular glycoprotein lacritin precursor are specific to lacrimal gland secretions.

Lacritin is an eye-specific growth factor that may play an important role in secretion and renewal of lacrimal and ocular surface epithelia. It is a secretory glycoprotein released apically from human lacrimal acinar cells. Lacritin also appears to be a product of meibomian gland [26]. Only salivary and possibly thyroid gland expresses lacritin, but at much lower levels. Lacritin is down regulated in the DES [32]. It functions as autocrine/paracrine enhancer of the lacrimal constitutive secretion, promoting sustained basal tearing, ductal cell mitogen and stimulator of corneal epithelial cells [33]–[34]. Thus tear proteins such as the lacritin can themselves act as regulators of tear secretion and as factors for renewal of ocular epithelia and down regulation of these protein can therefore contribute to the disease progression in terms of severity.

Immunoglobulin J (Ig J) is another protein that was down regulated in DES as observed in this study. Ig J plays critical role in increasing the antimicrobial activity of Ig A by combining the 2 monomeric Ig A chains to a polymeric form. Ig A is reportedly reduced in DES conditions [35]. Presence of Ig J chain in lacrimal gland tissue is reported [36]. However changes in this protein in tear fluid is not reported so far. We observed down regulation of Ig J in all types of DES associated with non Sjogrens, primary and secondary to RA. Studies showed down regulation of Ig J chain in salivary gland tissue of primary SS cases using proteomic approach after treatment with the steroids [37], [38]. Further quantification in tear would help in ascertaining the role in inflammation seen in DES especially before and after treatment.

The current study focuses on LPRR4. LPRR4 was found to be down regulated in maximum number of DES cases with maximum fold variation. As it was found to be a novel protein and not many studies are there, it was chosen for further validation. LPRR4 was found to be down regulated in DES cases irrespective of the cause of the dry eye syndrome, as seen by 2DE, in individual samples. DIGE was done in pooled sets of samples to further validate it. Quantitation of LPRR4 was done by ELISA to correlate it with the disease severity. A significant correlation was found between the levels of LPRR4 and the mild, moderate as well as the severe forms of DES. In our previous study, we identified LPRR4 as one of the significantly down regulated protein in DES [25]. This study revealed a significant reduction or absence of the LPRR4 protein in all types of dry eye syndrome associated with primary Sjogren’s syndrome, as well as secondary to rheumatoid arthritis apart from non Sjogren’s which included Steven Johnson’s syndrome. Reduction of this protein has been reported in Sjogren [26] as well as in blepharitis conditions [16]. This is the first report to state that decrease in LPRR4 is associated with all types of DES irrespective of the causative factor, since there is lacrimal gland involvement in all the types of DES studied. RA-DES showed the maximal decrease and this is probably associated with the number of severe cases in the group. This study also reports on the expression at protein level correlating clinically with the disease severity as evaluated by the Schirmer’s test and the TBUT test.

LPRR4 expression is reported in lacrimal acinar cells [39]. It is important to know the function of the protein to understand the relevance of this significant decrease in DES. However the structure function relationship of the protein is not yet elucidated. Most of the studies involving the structure and function have been done in the context of salivary gland. Salivary gland expresses proline rich proteins (PRPs) namely the basic proline rich proteins 1–4 and the acidic proline rich phosphoprotein [40]. Despite their overall similarity, the actual protein sequence of LPRR4 is significantly different from the salivary acidic PRRs based on the assessment of the sequence similarity. The mRNA expression of LPRR4 showed an ocular tissue specific expression in lacrimal gland when compared to corneal epithelium as seen in this study. However, the limitation of the study is that it is not possible to verify the changes in LPRR4 expression at the level of mRNA in the lacrimal gland of DES cases. Moreover it was not estimated in non DES conditions such as in keratitis and conjunctivitis. In MGD associated with DES there was a decrease in LPRR4 levels while in MGD without DES it was within the normal range (data not shown).

A protective function has been assigned for the salivary PRRs [41] in protecting the epithelial surfaces [42]. LPRR4 probably plays a similar protective role in the eye as a modulator of the bacterial flora either by promoting agglutination and clearance of bacteria or by promoting adherence of benign species to the epithelial surfaces thereby eliminating the binding of the other harmful ones [39]. A trend of increasing bacterial count with increase in grading of dry eye correlating with decrease in goblet cell density was reported in one of the study [43]. However, the study did not show any correlation between the increases in the bacterial count with that of the inflammation that warrants clinical intervention [43]. Binding to minerals or tannins may also be important for the protection of the ocular surfaces. The epithelial surface of the eye is exposed to an environment that contains tannins apart from microscopic mineral particles. LPRR4 seems to be an abundant tear protein and may therefore play a protective role.

Thus a significant down regulation of LPRR4 was observed in tear samples of dry eye condition and therefore based on this study it is proposed that, LPRR4 is a potential biomarker of DES. Further studies are required to understand the exact function of the protein in the protection of ocular surface.

## Supporting Information

Table S1 **Clinical details of DES patients for LPRR4 validation using ELISA.** The clinical parameters namely Schirmer’s, TBUT, diagnosis of DES, severity/grade of DES, fluorescence staining(FS), tear meniscus height (TMH), tear debris (TD), conjunctiva, cornea, lid and puncta status, systemic illness, symptoms and allergic reactions and the LPRR4 levels of the patients are given.
(DOC)
