# Supplementary material for: Lacrimal Proline Rich 4 (LPRR4) Protein in the Tear Fluid Is a Potential Biomarker of Dry Eye Syndrome
Source: PLoS One. 2012 Dec 18;7(12):e51979. doi: 10.1371/journal.pone.0051979 (PMC3525644; doi:10.1371/journal.pone.0051979)
Supplement: Table S1 — Clinical details of DES patients for LPRR4 validation using ELISA. The clinical parameters namely Schirmer’s, TBUT, diagnosis of DES, severity/grade of DES, fluorescence staining(FS), tear meniscus height (TMH), tear debris (TD), conjunctiva, cornea, lid and puncta status, systemic illness, symptoms and allergic reactions and the LPRR4 levels of the patients are given. (DOC) [file pone.0051979.s001.doc]

**Table S1: Clinical details of DES patients for LPRR4 validation using ELISA.**


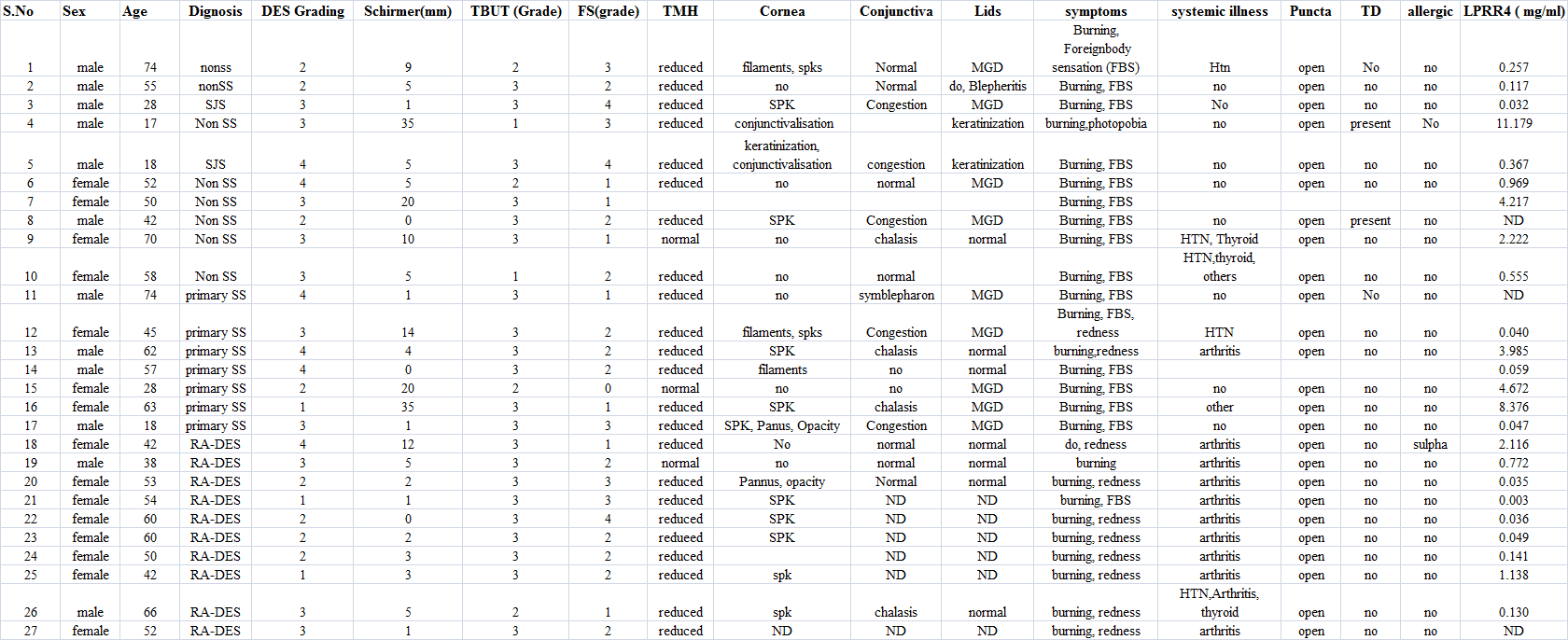


Note: TBUT grading - > 10 sec: grade 1, 5 – 10 sec: grade 2, < 5 sec grade 3 and 4.

Fluorescein staining grading- staining 1: grade 1, 2: grade 2, and 3, 4: grade 3 &4

DES grading – grade 1: mild, grade 2: moderate, grade 3 and 4: severe.

**Legend for Table S1:** **Clinical details of DES patients for LPRR4 validation using ELISA. The clinical parameters namely Schirmer’s, TBUT, diagnosis of DES, severity / grade of DES, fluorescence staining(FS), tear meniscus height (TMH), tear debris (TD), conjunctiva, cornea, lid and puncta status, systemic illness, symptoms and allergic reactions and the LPRR4 levels of the patients are given.**
